# Phase Transformation, Twinning, and Detwinning of NiTi Shape-Memory Alloy Subject to a Shock Wave Based on Molecular-Dynamics Simulation

**DOI:** 10.3390/ma11112334

**Published:** 2018-11-21

**Authors:** Man Wang, Shuyong Jiang, Yanqiu Zhang

**Affiliations:** 1 College of Mechanical and Electrical Engineering, Harbin Engineering University, Harbin 150001, China; wang_man28@hrbeu.edu.cn (M.W.); zhangyanqiu0924@sina.com (Y.Z.); 2 College of Materials Science and Chemical Engineering, Harbin Engineering University, Harbin 150001, China

**Keywords:** shape-memory alloy, NiTi alloy, molecular-dynamics simulation, shock loading, matrensitic transformation

## Abstract

Martensitic transformation, reverse martensitic transformation, twinning, and detwinning of equiatomic nickel–titanium shape-memory alloy (NiTi SMA) under the action of a shock wave are studied using a molecular-dynamics simulation. In the loading process of a shock wave, B2 austenite is transformed into B19′ martensite, whereas in the unloading process of the shock wave, B19′ martensite is transformed into B2 austenite. With repeated loading and unloading of the shock wave, martensitic transformation occurs along with twinning, but reverse martensitic transformation appears along with detwinning. The mechanisms for the twinning and detwinning of NiTi SMA subjected to a shock wave are revealed in order to lay the theoretical foundation to investigate the shape-memory effect and superelasticity.

## 1. Introduction

Shape-memory alloy (SMA) refers to a class of materials that are able to recover their original shape by heating to a certain critical temperature and are capable of possessing large recoverable elastic strains under unloading [1,2,3]. The former is called the shape-memory effect (SME), and the latter is called superelasticity (SE). Among SMAs, equiatomic or nearly equiatomic nickel–titanium (NiTi) SMAs are widely used in medical equipment and micro-electro-mechanical systems (MEMS) [4,5,6,7], owing to their excellent mechanical properties, SME, good biocompatibility, and wear and corrosion resistance [1]. 

In NiTi SMAs, SME and SE are attributed to the reversible temperature-induced and stress-induced transformation between B2 austenite and B19′ martensite [8,9,10,11], respectively. Compared to SME, the martensitic transformation in SE occurs at a higher temperature, usually above the austenite transformation finish temperature A_f_.

Over the past few decades, researchers have put a lot of effort into investigating the microstructure evolution mechanism related to the SME and SE in NiTi SMAs at the nanoscale. Based on experimental observations, Waitz et al. found that the smaller grain size contributes to lowering the transformation temperature of NiTi SMA, and that martensitic transformation is suppressed when grain size is less than a critical value [9,12]. Ahadi and Sun found that nanocrystalline NiTi SMA shows a sharp rise in the stress–strain response, while polycrystalline NiTi SMA with coarse grains exhibits an obvious plateau in the stress–strain response [10,13,14]. In addition, many researchers have shown great interest in the dynamic response of NiTi SMAs. Liao et al. studied the dynamic response of NiTi SMA under laser-shock peening (LSP), and they stated that higher laser intensity, as well as lower processing temperature, results in higher stress-induced martensite fraction [15]. Ye et al. studied the nanocrystallization of NiTi SMA by LSP and controlled annealing, and they found that a nanocrystalline structure with bimodal grains is formed at the top surface of the NiTi SMA, where the deformation twins are generated as well, and strength as well as ductility are substantially improved by means of LSP and controlled annealing [16].

Up to date, many experiments have been used to investigate the stress- and temperature-induced transformations of NiTi SMA. However, in situ experiments have a great limitation because the transformation temperature of nanocrystalline NiTi SMA is even able to reach 0 K [9]. As a tool for overcoming the aforementioned difficulties and supplementing the involved experiments, molecular-dynamics (MD) simulations are an effective method to understand the fundamental mechanisms at the atomic scale in detail. 

Kastner et al. performed a two-dimensional MD simulation for the martensitic transformation of NiTi SMA based on the Lennard–Jones potential model, where the simulation results were in good accordance with the experimental ones [17]. On the basis of the work by Kastner et al., Baimova et al. used Lennard–Jones potential to further investigate martensitic transformation of NiTi SMA by means of a two-dimensional MD simulation, where they obtained some valuable results [18,19,20,21]. Zhong et al. [22] modified the embedded-atom method Finnis–Sinclair (EAM-FS) potential established by Lai and Liu [23] to investigate the transformation and mechanical behaviors of NiTi SMA under compressive loading and unloading. They reported that, based on the nanoscale size effects, load serration, stress plateau, and large hysteresis loop in the stress–strain curve are attributed to the high stress, which is the driving force of the nucleation controlling phase transformation as well as deformation twinning in nanosized volumes. Mutter and Nielaba [24] also improved the potential developed by Lai and Liu [23] to investigate the stress-induced martensitic transformation at different temperatures. According to the second nearest-neighbor-modified embedded-atom method (2NN MEAM), Ko et al. optimized a NiTi potential and accurately predicted the temperature- and stress-induced transformation in equiatomic NiTi SMA [25]. Srinivasan et al. [26] compared the 2NN-MEAM potential with the EAM-FS one, and found that the 2NN-MEAM potential more accurately predicts elastic constants and captures SE behavior. At present, most of the MD simulations of NiTi SMAs focus on temperature- and stress-induced transformations under static loading [22,27,28,29,30], but rarely involve the study of phase transformation and mechanical behaviors under high strain-rate impact load.

In particular, from the viewpoint of time scale, shock loading is more suitable for investigation by MD simulation [31], where the classical equation of motion can be numerically solved for per atom in the system [32]. In the present study, our novel wok is based on the fact that martensitic transformation, reverse martensitic transformation, twinning, and detwinning of equiatomic NiTi SMA under the action of a shock wave are studied via molecular-dynamics simulation. Furthermore, the mechanisms for twinning and detwinning of NiTi SMA subjected to the shock wave are revealed in order to lay the theoretical foundation for investigating the shape-memory effect and superelasticity.

## 2. Modeling and Methods 

### 2.1. MD Model

**Figure 1:**
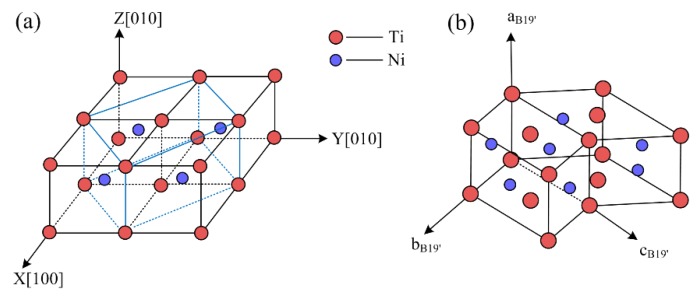
Crystal structures of nickel–titanium shape-memory alloy (NiTi SMA): (**a**) B2 cubic; (**b**) B19′ monoclinic.

In the present study, equiatomic NiTi SMA was applied to all the MD simulations. The high-temperature parent phase of the alloy is austenite phase, and its crystal structure is B2 cubic structure (as illustrated in Figure 1a), whereas the low-temperature phase of the alloy is the martensite phase, and its crystal structure is B19′ monoclinic structure (as indicated in Figure 1b). 

**Figure 2:**
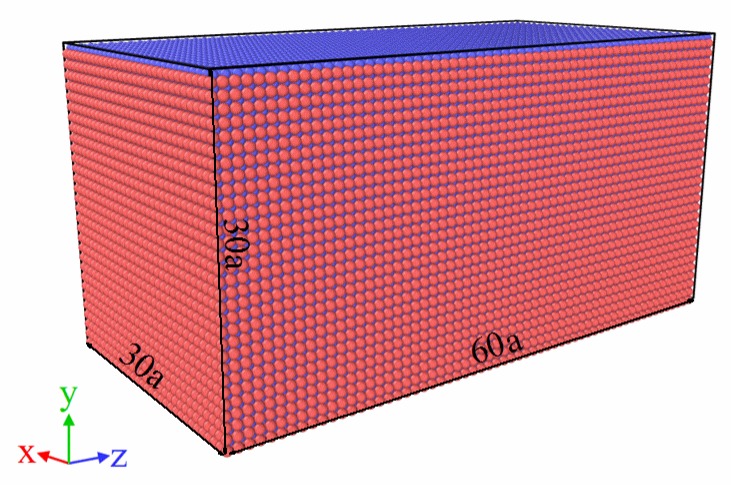
Initial structure of the molecular-dynamics (MD) model with a B2 cubic phase containing 108,000 atoms.

The initial structure of the MD model belongs to B2 cubic phase and possesses dimensions of 30a × 30a × 60a, where a is the lattice constant and it is equal to 2.999 Å, as is shown in Figure 2. In addition, 108,000 atoms are contained in the MD model. Furthermore, the three co-ordinate axes, x, y and z, correspond to the [100], [010], and [001] orientations, respectively.

### 2.2. Interatomic Potentials

The key to an MD simulation is to properly select the interaction potential between atoms, which controls the interaction behavior between atoms and determines the fundamental properties of materials. In our work, all MD simulations were performed according to the 2NN-MEAM potential developed by Ko et al. [25]. The 2NN-MEAM potential has high prediction accuracy for the superelastic behavior, transformation behavior, and Young’s modulus of the NiTi SMA. Ko et al. [25] optimized the pure Ni and Ti systems that were developed by Lee et al. [33] and Kim et al. [34], respectively, by using a force-matching method [35], and then they developed the 2NN-MEAM potential of NiTi SMA. The involved parameters of the potential of NiTi SMA were optimized by Ko et al. [25], and they are listed in Table 1. The parameters are described as follows: $\Delta E_{f}$ represents the enthalpy of formation in the reference structure (B2 NiTi), and its unit is eV/atom. $E_{c}$ stands for the cohesive energy, and its unit is eV/atom. $r_{e}$ denotes the equilibrium nearest-neighbor distance, and its unit is Å. B is the bulk modulus and its unit is 10^12^ dyne/cm^2^. C is the screening parameter of B2 austenite, and d is the adjustable parameter.

### 2.3. Simulation Software

MD simulation of the NiTi SMA under the action of a shock wave was implemented via large-scale atomic/molecular massively parallel simulator (LAMMPS, 23 October 2017 version, Albuquerque, NM, USA) [36]. Scientific visualization OVITO software (2.9.0, Darmstadt, Germany) [37] was used to analyze the atomistic simulation results. To classify the local structural environment of the particles, the polyhedral template-matching (PTM) method [38] was used because it has higher reliability [39] in the presence of strong thermal fluctuations and strain. Furthermore, it can directly calculate local orientation, elastic deformation, and strain and alloy type [37]. Although the PTM method is originally not designed to distinguish between B2 and B19′ phases, it has been confirmed that it is well suitable for that purpose [28,30]. In other words, the PTM method cannot recognize a B19′ monoclinic structure, so a hexagonal close-packed (HCP) structure is defaulted as the B19′ monoclinic structure.

### 2.4. Shock Loading Conditions

The MD simulation of the temperature-induced transformation of NiTi SMA was firstly carried out to determine the temperature where the shock wave is implemented. As a consequence, the four typical phase-transformation temperatures, including martensite-transition starting-temperature M_s_, martensite-transition finishing-temperature M_f_, austenite-transition starting-temperature A_s_, and austenite-transition finishing-temperature A_f_, are obtained. The time step of all MD simulations was defined as 0.001 ps. Periodic boundary conditions were used along all the three dimensions. Firstly, B2 structure is equilibrated at 550 K under NPT ensemble for a sufficiently long time. Then, temperature was gradually reduced to 50 K and subsequently enhanced to 550 K at the cooling and heating rates of ±0.5 K/ps. Finally, the MD simulation recorded the variation of atomic volume caused by temperature so as to capture the phase-transformation temperatures. 

**Figure 3:**
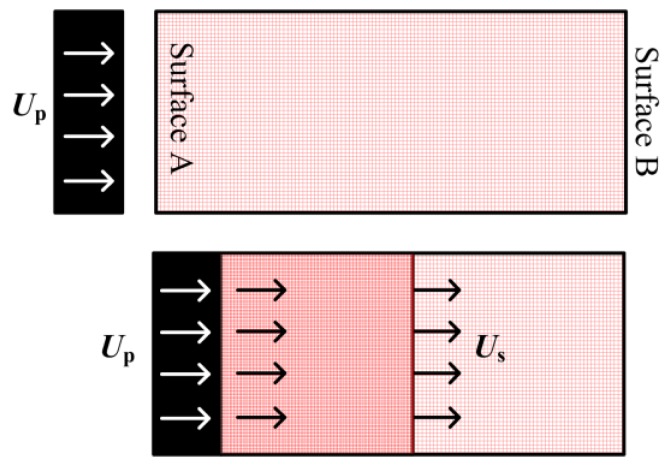
Schematic representation of shock-wave propagation.

It was crucial to produce a shock wave for the purpose of performing an MD simulation during the shock loading of NiTi SMA. Here, the “piston impact method” [32,40,41,42] was used to create a shock wave owing to its similarity to experiment, especially LSP experiments. The MD shock simulations were set up by launching a piston toward a stationary target at an Up velocity, which would stop after collision, where Up is the “piston velocity”. In addition, the piston frequently possessed the same material as the target [31], which provided an easier way to program for MD shock simulation. The schematic representation of shock-wave propagation is shown in Figure 3. It is noted that, when a shock wave reaches a free surface, it behaves according to the impedance rules of a wave traveling through different environments [43], which result in the reflection of a compressive shock wave. Therefore, the compressive shock wave becomes tensile after reaching the free boundary. These tensile waves are also called rarefaction, unloading, release, or decompression waves. In the present study, four stages were simulated during the propagation of the shock wave. The shock wave travels from Surface A to Surface B at the first stage (Stage I), whereas the shock wave returns from Surface B to Surface A at the second stage (Stage II). Subsequently, the shock wave again travels from Surface A to Surface B at the third stage (Stage III), and, similarly, the shock wave returns from Surface B to Surface A at the fourth stage (Stage IV). Stages I and II can be viewed as a complete cyclic process of the shock wave. In the same manner, Stages III and IV can be regarded as another complete cyclic process of the shock wave. However, in fact, the four stages arise continuously during the propagation of the shock wave.

**Figure 4:**
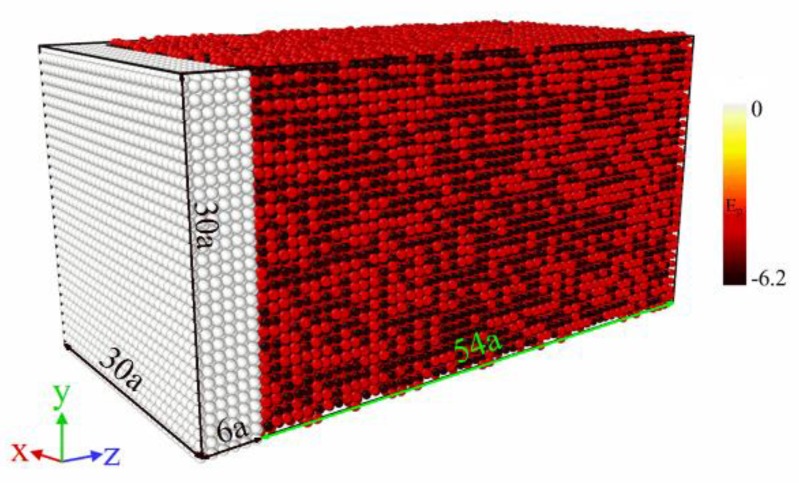
MD simulation model of piston impact loading.

Shock loading is exerted at temperature T above A_f_ so as to reveal the phase-transition mechanism of NiTi SMA under the action of a shock wave. Based on the molecular-dynamics model as shown in Figure 2, a total thickness of 6a, which approximates to a few layers of atoms, was set as the impact piston, and atomic potential energy E_p_ was colored by using OVITO software to distinguish between the piston and the target; the gray part on the left side represents the piston, and the other represents the target, as shown in Figure 4. Initially, the system was equilibrated at temperature T under the NPT ensemble with periodic boundary conditions in all dimensions. Subsequently, the normal surfaces to the propagation direction of the shock wave were reset as shrink-wrapped boundary conditions to always encompass the atoms in that dimension. Then, the ensemble was reset as NVE to ensure an adiabatic environment during impact loading. The shock wave was produced by driving a piston with a velocity of Up = 1.0 km/s along the [001] direction. For the purpose of tracking the propagation process of the shock wave in detail, bins were defined along the [001] orientation, where the thickness of each layer is 3 Å. The atoms in each layer were averaged so as to obtain the involved thermodynamic parameters. The simulation process ran 20,000 time steps, which were long enough for the purpose of getting the entire shock-wave trajectory.

## 3. Results and Discussion

### 3.1. Determination of Shock Temperature

**Figure 5:**
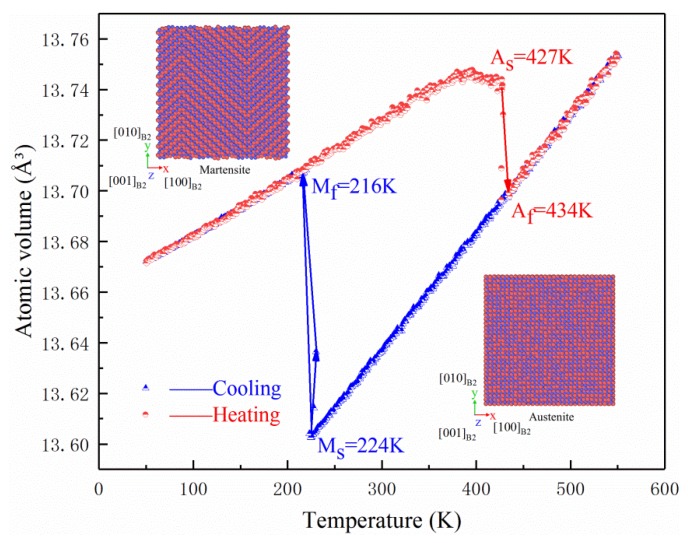
Variation of atomic volume with temperature in the MD simulation of the temperature-induced martensitic transformation of NiTi SMA.

**Figure 6:**
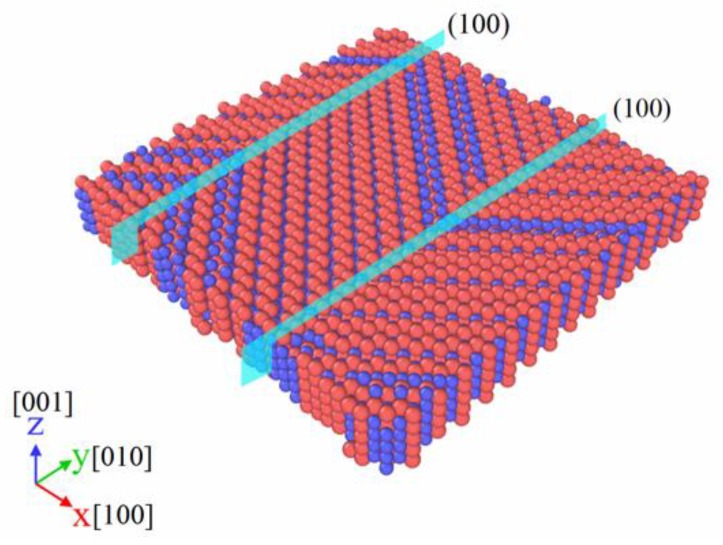
Crystal structure of martensitic twins with a (100) twin plane.

Figure 5 indicates the variation in atomic volume with temperature during the temperature-induced transformation of NiTi SMA between B2 and B19′ on the basis of the molecular-dynamics simulation. It is noted in Figure 5 that, when atomic volume is abrupt, it means that phase transformation occurs. During the cooling process from 550 to 50 K, martensitic transformation occurs. When the temperature is back to 550 K, the reverse martensitic phase transformation occurs. The corresponding phase-transformation points can be obtained as: A_s_ = 427 K, A_f_ = 434 K, M_s_ = 224 K, M_f_ = 216 K. In addition, in the martensitic transformation process, it is found that the formation of martensitic twins is accompanied by the (100) twin plane, as illustrated in Figure 6. According to the aforementioned simulation results, 450 K was selected as the temperature where the molecular-dynamics simulation of NiTi SMA under the action of the shock wave was implemented. It is apparent that, at that temperature, NiTi SMA is in a fully B2 austenitic state.

### 3.2. Propagation of Shock Wave

**Figure 7:**
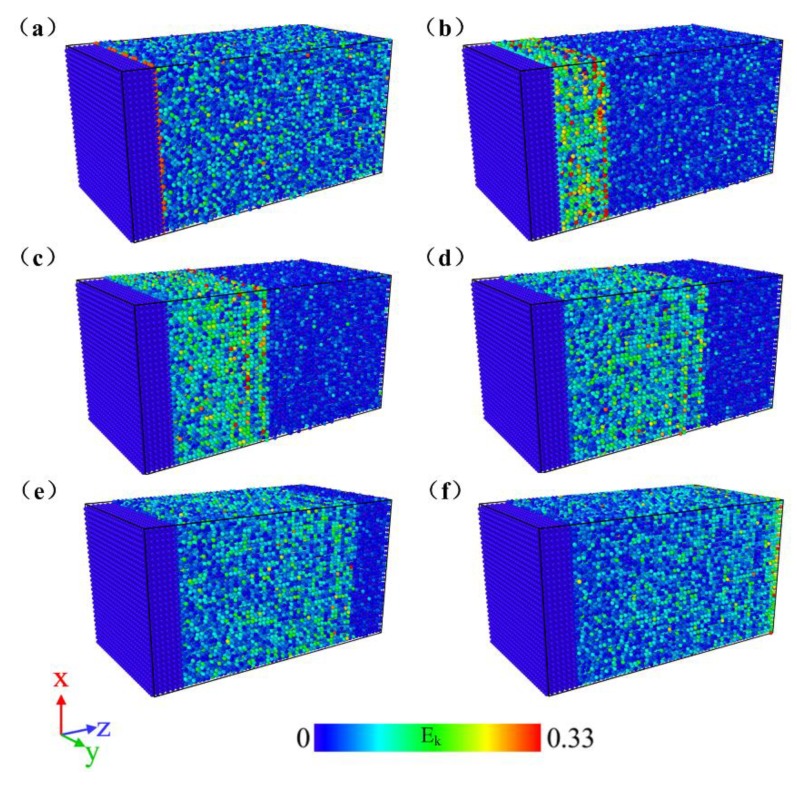
Propagation trajectory of the shock wave in NiTi SMA: (**a**) t = 0.0 ps; (**b**) t = 0.6 ps; (**c**) t = 1.2 ps; (**d**) t = 1.8ps; (**e**) t = 2.4 ps; (**f**) t = 3.0 ps.

Figure 7 shows the propagation trajectory of the shock wave based on MD simulation at Stage I (0–3 ps). When 3000 time steps (i.e., 3 ps) were run, the shock wave traveled from the front end of the block (Surface A) to the end (Surface B). In the simulation process, kinetic energy per atom was colored by OVITO software to record the propagation process of the shock wave, where E_k_ represents the kinetic energy per atom.

**Figure 8:**
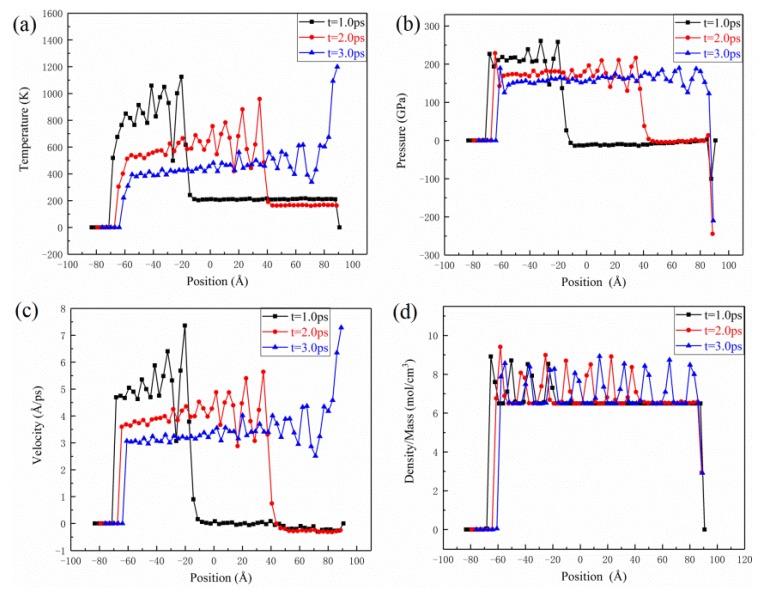
Variation of average value of per atom in all physical quantities during propagation of the shock wave: (**a**) temperature; (**b**) pressure; (**c**) velocity; (**d**) ratio of density to mass.

In the shock-wave loading process, materials were compressed at an ultra-high rate, which resulted in a series of variations of physical quantities, such as temperature, pressure, velocity, volume, and density. Therefore, it was of great significance to investigate the variations of the involved physical quantities during the propagation of the shock wave. However, in the present work, emphasis was on the transformation and mechanical behaviors of NiTi SMA during the propagation of the shock wave, so the physical quantities were given only at Stage I, where the shock wave took 3 ps to travel from Surface A to Surface B. Figure 8 indicates the variation of physical quantities with the propagation distance of the shock wave, where all physical quantities are based on the average value of per atom. From Figure 8c, we can see that the impact between piston and target was found to quickly occur, which caused a sharp rise in pressure and temperature, as indicated in Figure 8a,b, respectively. The location of the shock-wave front at various times is identified in Figure 8d.

### 3.3. Martensitic Transformation and Reverse Martensitic Transformation

**Figure 9:**
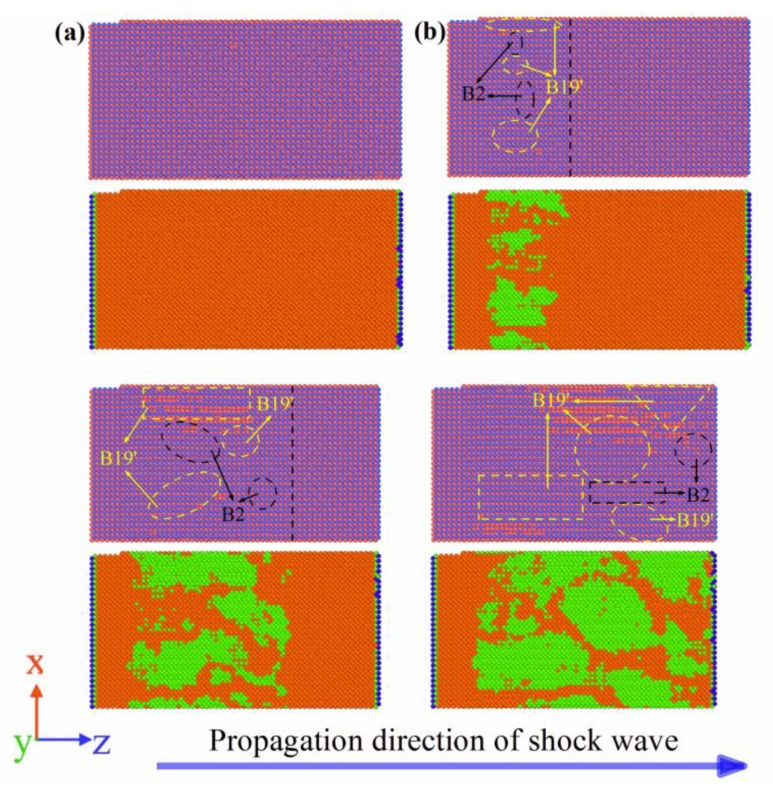
Martensitic transformation at Stage I: (**a**) 0.0 ps; (**b**) 1.0 ps; (**c**) 2.0 ps; (**d**) 3.0 ps. (orange and green represent the B2 phase and B19′ phase, respectively, but blue represents the other phase).

Figure 9 shows the MD simulation results of NiTi SMA at Stage I during the propagation of the shock wave. It can be seen from Figure 9 that, under the action of the shock wave, B2 austenite was converted to B19′ martensite. Furthermore, with the progression of the shock wave, the volume fraction of the martensite phase continuously increased. When the simulation time was 3 ps, the front of the shock wave arrived at Surface B. At the time, martensitic transformation completely finished. As a consequence, the martensite phase covered 61% of the NiTi SMA by volume fraction. In other words, B2 austenite was not totally transformed into B19′ martensite.

**Figure 10:**
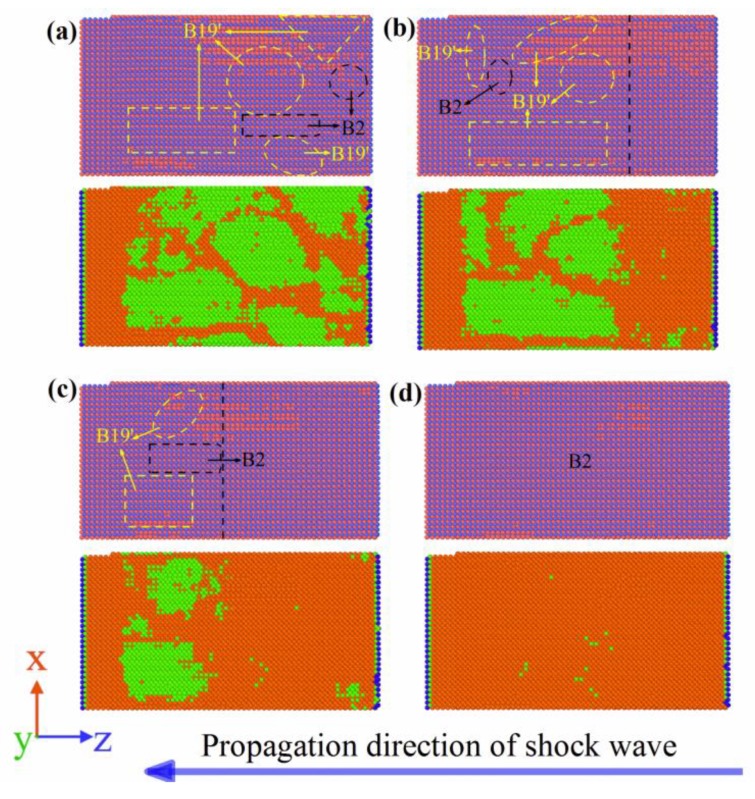
Reverse martensitic transformation at Stage II: (**a**) 3.0 ps; (**b**) 4.0 ps; (**c**) 5.0 ps; (**d**) 6.0 ps. (orange and green represent B2 phase and B19′ phase, respectively, but blue represents the other phase).

Figure 10 shows the MD simulation results of the NiTi SMA at Stage II during the propagation of the shock wave. It can be proposed that Stage II corresponds to the unloading process of the shock wave, where the reverse martensitic transformation occurs. In other words, the B19′ martensite phase was transformed into the B2 austenite phase. Furthermore, with the progression of the shock wave, the martensite phase gradually decreased. When the simulation time was 6 ps, the front of the shock wave arrived at Surface A. At this time, reverse martensitic transformation completely finished. However, it can be observed that an extremely small amount of the martensite phase, which is about 2.2% by volume fraction, remained in the NiTi SMA. That is to say, the B19′ martensite phase was not completely transformed into B2 austenite phase.

In the present work, the martensitic transformation of the NiTi SMA that was induced by shock wave stems from the stress-induced one. The mechanical behavior of the NiTi SMA is closely related to the transformation behavior. Many researchers have confirmed that the stress-induced martensitic transformation of the NiTi SMA can occur during tension, compression, and shear loading [44,45,46]. Furthermore, the strain rates substantially influence the transformation behavior, and thus the mechanical behavior of the NiTi SMA [47]. The stress–strain curve of the NiTi SMA generally possesses a plateau representing stress-induced martensitic transformation during loading at a quasistatic strain rate. However, the stress plateau gradually disappears with an increasing strain rate, which has been confirmed by Nemat-Nasser S. et al. [48,49]. Wang et al. provided direct experimental evidence that stress-induced martensite transformation occurs in NiTi SMA undergoing laser-shock peening, where the strain rate is approximately 10^7^ s^−1^ [50,51]. It is concluded that the mechanism of stress-induced martensite transformation at an ultra-high strain rate is obviously different from that at a low strain rate. In our work, the mechanism of stress-induced martensite transformation and reverse martensite transformation at an ultra-high strain rate is revealed based on the MD simulation in terms of the atomic scale, which is very significant for investigating SME as well as SE under shock-loading conditions.

### 3.4. Twinning and Detwinning

**Figure 11:**
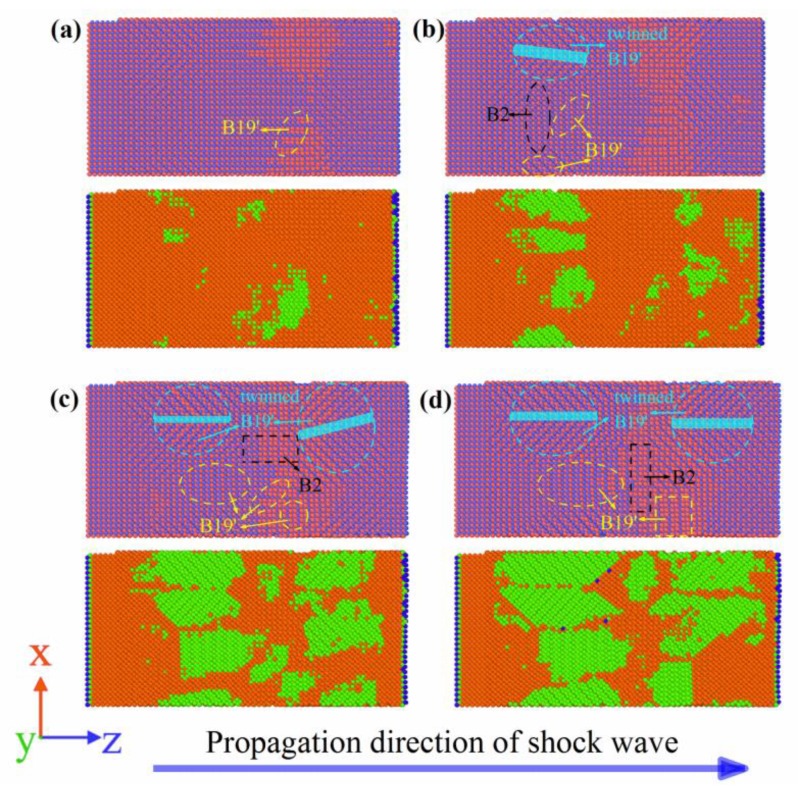
Martensitic transformation and twinning at Stage III: (**a**) 6.0 ps; (**b**) 8.2 ps; (**c**) 9.2 ps; (**d**) 10.2 ps. (Orange and green represent the B2 phase and B19′ phase, respectively, but blue represents the other phase).

Figure 11 shows the MD simulation results of the NiTi SMA at Stage III during the propagation of the shock wave. Similar to Stage I, under the action of shock wave, the B2 austenite phase was converted to the B19′ martensite phase. However, compared to Stage I, martensitic twins occur in Stage III. In addition, with the progression of the shock wave, martensitic twins with a noncoherent interface were gradually converted to martensitic twins with a coherent interface. When the simulation time was 10.2 ps, the front of the shock wave arrived at Surface B. At that time, martensitic transformation was completed. As a consequence, the martensite phase occupies 45% of the NiTi SMA by volume fraction. In the same manner, B2 austenite was not totally transformed into B19′ martensite.

**Figure 12:**
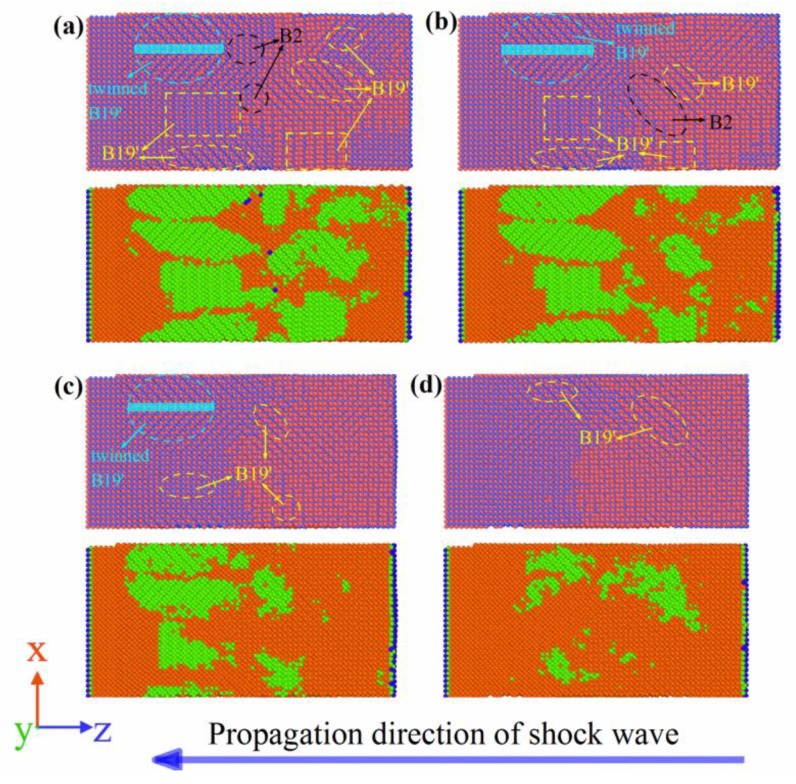
Reverse martensitic transformation and detwinning at Stage IV: (**a**) 10.2 ps; (**b**) 11.4 ps; (**c**) 12.8 ps; (**d**) 14.2 ps. (Orange and green represent the B2 phase and B19′ phase, respectively, but blue represents the other phase).

Figure 12 shows the MD simulation results of the NiTi SMA at Stage IV during the propagation of the shock wave. Similar to Stage II, Stage IV corresponds to the unloading process of the shock wave, where B19’ martensite was transformed into B2 austenite. In particular, as reverse martensitic transformation occurred, the coherent interface of martensitic twins was gradually destroyed. This means that detwinning took place during the reverse martensitic transformation of the NiTi SMA. When the simulation time was 14.2 ps, the front of the shock wave arrived at Surface A. At that time, reverse martensitic transformation completely finished and the martensitic twins completely disappeared. Compared to Stage II, the retained martensite phase was approximately 10% in Stage IV.

Many experimental results have shown that three kinds of typical martensitic twins, Type I and II twins, and (001) or (100) compound twins, are frequently observed in NiTi SMA [4,52,53,54]. In general, Type I and Type II twins satisfy phenomenological crystallographic theory, so they possess a characteristic of lattice invariant shear. However, (100) compound twins are not suitable for phenomenological crystallographic theory and, hence, are not characterized by a lattice invariant shear. Therefore, (100) compound twins are generally viewed as deformation twins [4,52,53,54].

In the present work, (100) compound twins are formed in the NiTi SMA subjected to a shock wave, whereas Type I and type II twins were not observed. It is not excluded that the phenomenon probably resulted from the limitation of interaction potential. In other words, the present potential is probably not suitable for investigating Type I and type II twins. However, this does not affect the topic of the manuscript with respect to the twinning and detwinning of the NiTi SMA under the action of a shock wave. It is well known that twinning and detwinning play a considerable role in the SME and SE of NiTi SMA. In addition, (100) compound twins are fairly complicated. Although (100) compound twins are generally regarded as a result of deformation twinning, they are obviously different from conventional deformation twins. Ordinary deformation twinning, also called mechanical twinning, usually occurs during the plastic deformation of metal materials, and it is different from transformation twinning, which frequently takes place during martensitic transformation [55]. In particular, ordinary deformation twinning is easier to appear in a cryogenic environment or at high strain rates, and it generally changes the crystal orientation rather than the crystal structure. However, (100) compound twins frequently occur during the phase transition from B2 to B19’ during heat treatment [53,54]. It cannot be denied that the occurrence of (100) compound twins is related closely to thermal–mechanical processing history [53,54]. P. Šesták has stated that, according to the first-principle calculation, (100) compound twins can substantially stabilize the B19′ martensite phase of NiTi SMA [56]. T. Ezaz et al. calculated the energy barriers for (100) and (001) compound twins based on first-principle calculation, and they found that the former is much higher than the latter [57]. In our work, (100) compound twins occur along with the stress-induced martensite transformation in the NiTi SMA subjected to the shock wave. Therefore, a discussion on whether the (100) compound twins belong to deformation twins or transformation twins is out of the scope of this manuscript. This manuscript focuses on the evolution of the twinning and detwinning of the NiTi SMA subjected to a shock wave, which lays the theoretical foundation for investigating the SME and SE of NiTi SMA. In fact, Type II twins are observed more frequently in NiTi SMA, and it has a predominant influence on the SME of NiTi SMA. Furthermore, many researchers have deeply investigated the detwinning of Type II twins [58,59,60]. In particular, Dilibal S captured the detwinning mechanism of NiTi SMA by using advanced digital-image correlation (DIC) technology [61]. Therefore, it seems that it is more significant to elucidate the detwinning mechanism of Type II twins by means of MD simulation. However, this would be difficult to realize for a while due to the complexities of the twins as well as the limitations of potential and MD modeling for NiTi SMA. It is a promising perspective for NiTi SMA to develop novel interaction potential and to establish a new MD model in the future.

## 4. Conclusions

The martensitic transformation, reverse martensitic transformation, twinning, and detwinning of the NiTi SMA subjected to a shock wave were investigated on the basis of an MD simulation. The following conclusions could be drawn according to the four stages of the shock wave.

(1) In Stage I, namely, the loading process of the shock wave, B2 austenite was transformed into B19’ martensite. In Stage II, namely, the unloading process of the shock wave, reverse martensitic transformation occurred, where the B19′ martensite phase was converted to the B2 austenite phase. In addition, twinning and detwinning were not observed in these two stages.
(2) In Stage III, namely, the second loading process of the shock wave, B2 austenite was transformed into B19′ martensite, and, simultaneously, martensitic twins occurred. Furthermore, with the progression of the shock wave, noncoherent twin boundaries were gradually converted to coherent ones. In Stage IV, namely, the second unloading process of the shock wave, reverse martensitic transformation occurred, where B19′ martensite was transformed into B2 austenite and, simultaneously, detwinning took place.
(3) (100) compound twins occurred along with stress-induced martensite transformation in the NiTi SMA subjected to the shock wave, whereas Type I and Type II twins were not observed. This is probably due to the limitation of interaction potential of NiTi SMA. It is a promising perspective for NiTi SMA to develop novel interaction potential and to establish a new MD model in the future.

## Figures and Tables

**Table 1:** Optimized second nearest-neighbor-modified embedded-atom method (2NN-MEAM) potential parameters for the NiTi system.

$\Delta E_{f}$	$E_{c}$	$r_{e}$	B	d	$\rho_{0}^{Ni}:\rho_{0}^{Ti}$	$C_{\left(Ni-Ti-Ni\right)}$		$C_{\left(Ti-Ni-Ti\right)}$		$C_{\left(Ni-Ni-Ti\right)}$		$C_{\left(Ti-Ti-Ni\right)}$	
						min	max	min	max	min	max	min	max
–0.36	4.96	2.612	1.2818	0.025	1:1	0.25	1.7	0.09	1.7	0.49	1.4	1.6	1.7
